# An Exploration of U.S. Southern Faith Leaders’ Perspectives of HIV Prevention, Sexuality, and Sexual Health Teachings

**DOI:** 10.3390/ijerph17165734

**Published:** 2020-08-08

**Authors:** Latrice C. Pichon, Terrinieka Williams Powell, Andrea Williams Stubbs, Nicole Becton-Odum, Siri Ogg, Trisha Arnold, Idia B. Thurston

**Affiliations:** 1 School of Public Health Division of Social and Behavioral Sciences, The University of Memphis, Memphis, TN 38152, USA; sirib@casimirtrials.com; 2 Bloomberg School of Public Health, Johns Hopkins University, Baltimore, MD 21205, USA; terri.powell@jhu.edu; 3 Department of Infectious Diseases, St. Jude Children’s Research Hospital, Memphis, TN 38105, USA; Andrea.Stubbs@STJUDE.ORG; 4 Nicole Becton Ministries Restoring Lives Through Christ, Memphis, TN 38109, USA; bectonn@bellsouth.net; 5 Department of Psychology, The University of Memphis, Memphis, TN 38152, USA; trisha_arnold@brown.edu; 6 Department of Psychological and Brain Sciences Texas A & M University and School of Public Health, Texas A & M Health, College Station, TX 77843, USA; idiathurston@tamu.edu

**Keywords:** religion and health, HIV/AIDS, CBPR, church-based health promotion, focus groups

## Abstract

Reducing human immunodeficiency viruses (HIV) and acquired immune deficiency syndrome (AIDS) racial/ethnic disparities in the Deep South has been a critical objective of the U.S. National HIV/AIDS Strategy. This finding, originally published in 2010 by the Office of National AIDS Policy, serves as a complement to the Health and Human Resources and Services Administration’s Ending the HIV Epidemic (EtHE): A Plan for America. The EtHE plan, released in 2019, emphasizes community stakeholder involvement to achieve the planning goals of decreasing new HIV infections in key U.S. geographic areas. According to the plan, an important stakeholder is faith leaders, especially around stigma reduction. This paper focuses on a community–academic research partnership’s exploration of southern Black faith leaders’ teaching perspectives regarding HIV prevention, sexuality, and sexual health in predominantly Black congregations in Memphis, Tennessee. The partnership conducted four focus groups using a semi-structured discussion interview. Any adult faith leader involved in ministry work in a predominantly Black church was eligible to participate in the discussion. A total of 26 faith leaders with a mean age of 54, representing four Christian denominations, consented to participate in the study. Emerging themes included: (1) restriction of scripture to teach prevention and address sexuality, (2) role of secrecy and silence in living with HIV, and (3) impact of the stigma of HIV and sexuality. Findings may inform nationwide jurisdictional implementation plans, particularly for faith-based interventions in southern churches working toward ending the HIV epidemic.

## 1. Introduction

Despite making up only 2% of the U.S. population, Black men who have sex with men (BMSM) account for 75% of all new HIV infections [1]. While just over a third of the U.S. population lives in the southern United States, more than 50% of HIV incidence is reported from this region [2]. Tennessee (TN) is in the Deep South, a subset of nine southern states driving the HIV epidemic, where 27.8% of newly diagnosed BMSM were between 15–34 years of age [3]. A high proportion of newly diagnosed persons are concentrated in a few geographic regions of the state, including the city of Memphis (study site). Rates of HIV infection in Memphis increased between 2014–2018 [3]. Memphis ranks third among all U.S. metropolitan statistical areas (MSA) for new HIV infections and has been identified in the U.S. Ending the HIV Epidemic (EtHE) Initiative: A Plan for America as a high HIV burden geographic area of focus [4].

The EtHE Initiative calls upon all sectors to work toward addressing four pillars that can significantly impact the HIV epidemic (i.e., diagnose, HIV care and treatment, prevent, respond) [4]. To achieve the EtHE goal of ending the HIV epidemic in the U.S. by 2030, success depends on engaging the faith community to promote HIV awareness, provide HIV prevention education, normalize HIV testing, and reduce HIV-related stigma. Contextual factors are important considerations when conducting faith-based HIV prevention. Research demonstrates that 85% of Blacks report religion as being very important to them, and more than half of Blacks report attending religious services at least once a week [5]. Moreover, 64% of Blacks living in the South are members of Historically Black Churches [5]. Thus, faith, religion, spirituality, and the church play a critical role in the lives of Black Americans, especially in the Deep South. Specific to young BMSM, studies show as much as 50% of participants report attending church once a month or more [6].

The field of faith-based HIV prevention research has evolved considerably [7,8]. The robust literature demonstrates churches are providing congregations and their surrounding community access to HIV education, prevention, and testing, which often is informed by biblical principles [9,10]. There are a number of factors facilitating readiness to address HIV, including having the blessing and support of the church leadership, purview on sexual health, and resources to implement prevention [11,12]. Yet a notably, well-documented social challenge remains—stigma—which disrupts people living with HIV from accessing care and serves as a threat to public health [13,14,15,16,17,18,19]. One area that faith leaders and researchers continue to grapple with is how best to reconcile sexuality and HIV with biblical teachings. For churches to fully engage in HIV prevention messaging, faith leaders (e.g., Senior Pastor, Ministerial Team) may need to consider and be open to discussing sexuality and same sex relationships, especially as it relates to stigma and mental health functioning among Black communities.

While human sexuality, by definition, is not limited exclusively to homosexuality, in faith contexts, it often is used interchangeably [20]. Recent findings from an ethnographic study explored the ideologies of homosexuality on HIV prevention with pastors ministering to predominantly Black congregations in Milwaukee, WI [21]. This study revealed pastors expressed messages of love, tolerance, and acceptance, but still struggle with the belief that homosexuality is a sin [21]. In another study, BMSM participated in in-depth interviews to inform strategies to address HIV prevention efforts in Baltimore, MD. One of the recommended strategies identified was reducing homosexuality related stigma by increasing interpersonal and institutional acceptance in churches [6]. Given the growing and alarming HIV disparities among BMSM, additional explorations are needed to understand the role of churches.

Churches serving predominantly Black congregations and communities remain one of the last standing and stable institutions in many Black communities. Churches can be a trusted source of health information, especially when congruent with individuals’ own beliefs [22]. Understanding the extent to which churches are prepared to address HIV associated risk behaviors, stigma, and testing needs within their congregations is a necessary antecedent to designing appropriate interventions for church settings [11]. Learning how to engage churches and faith leaders in discussions about sensitive aspects of HIV prevention is key to decreasing HIV incidence and keeping the community viral load down, tasks that are critical to making an impact along the HIV continuum of care [23].

The updated National HIV/AIDS Strategy (NHAS) (pre-cursor to EtHE) has four primary goals, including to reduce HIV incidence, increase access to care, improve health outcomes, and reduce HIV disparities in the South [24,25]. The EtHE initiative complements the goals of the NHAS Strategy by requiring collaborative and coordinated partnerships, including the faith community, to address core pillars [4]. Taken together, both national strategies highlight opportunities to explore threats to HIV resurgence. This paper discusses faith leaders’ teaching perspectives of HIV prevention, sexuality, and sexual health in their predominantly Black congregations. The goal is to identify potential barriers to inform the development of effective interventions in churches and local Ending the Epidemic implementation plans.

## 2. Materials and Methods

### 2.1. Design, Recruitment, and Sample

This study employed a descriptive, qualitative design to gain a better understanding of teaching perspectives of HIV prevention, sexuality, and sexual health among a convenience sample of faith leaders pastoring predominantly Black congregations in Memphis, Tennessee. Faith leaders are defined as any member of the ministerial staff appointed by the senior pastor, such as the senior pastors themselves, youth minister, and first lady. A participatory research approach was employed by involving all community–academic partners to address the study objectives, and the project was conceptualized by the community [26]. This partnership consisted of a non-profit, faith-based community organization, an HIV prevention coalition, and a university. Data collection and recruitment involved both community and academic partners. Finally, data were interpreted and prepared for publication using collaborative processes with two community partners included as co-authors [27].

Multiple strategies to recruit faith leaders were employed, including personal and professional networks. A publicly available e-directory compiling addresses and telephone numbers of approximately 300 Memphis area traditional Black churches was reviewed, and data were extracted. Study announcements were made at local public events. Distribution of study advertisements occurred at churches, community events, health centers, and the local ministerial alliance.

Faith leaders involved in ministry work at a predominantly Black church who self-reported being at least 18 years of age were eligible to join the discussion group. These included pastors, ministry leaders, and other leaders (i.e., youth advisor, assistant health and church clerk, Aides for HIV/AIDS Networking to Guide and Educate Lives (A.N.G.E.L.)) appointed by the pastor. All participants had to have the authority to make decisions about health ministry activities for the church. The University of Memphis Institutional Review Board (PRO-FY 2359) approved the research.

### 2.2. Data Collection

Four focus groups (FG) were facilitated by our trained community collaborator (fourth author), who serves as a Health and Wellness Minister and has engaged over 40 churches in HIV prevention. The size of the groups ranged from 5 participants (FG1, FG2, FG3) to 11 participants (FG4). Two groups were stratified by gender, but not faith leader role. All leaders actively participated, and the facilitator ensured each participant had an opportunity to speak with consideration to the potential variations in hierarchy levels of faith leaders. The first author (study principal investigator) trained her to facilitate the discussions and to conduct the informed consent process. Additionally, the first author took copious notes and audiotaped the discussion to ensure that all verbal and non-verbal forms of communication were captured [28]. Each focus group lasted approximately two hours and was held at a faith-based community health center.

We used a semi-structured focus group interview, in which participants were asked about how sexuality and sexual intercourse were discussed in their church [29]. The interview guide was informed, vetted, and approved by the community–academic partnership. Discussion questions included: “How, if at all, does your church address issues of sex? sexuality? homosexuality?”, “What types of messages do you share with members of your congregation about sex, sexuality?”

All participants completed a brief survey following the informed consent process and before the focus group discussions began. The survey included demographic questions (e.g., gender, age, education, marital status), denominational affiliation (e.g., Baptist, Methodist, non-denominational), their leadership role in the church they represented, and comfort level discussing sexual health content areas with adults and youth congregational members. To assess comfort level, participants were asked about each content area using the stem, “How comfortable are you discussing the following with the youth and/or adults in your church/faith-based organization?” The topics included sexual behaviors, such as anal, oral, and vaginal sex, sexuality, and condom use. Response options ranged from 1 to 4, where 1 represented not comfortable, and 4 signified very comfortable. Faith leaders were compensated with a $40 gift card for their time.

### 2.3. Analysis

Focus group discussions were transcribed verbatim. Using the research question as a guide, the community and academic partners took a deductive thematic approach to develop the codebook. There were 39 codes in the final codebook. To begin the coding process, each transcript was coded independently by two members of the research team [30]. Next, the two researchers discussed the categories each had independently identified through open coding during their regularly scheduled weekly meetings. The weekly meetings were convened to ensure the trustworthiness and dependability of the research findings [31,32]. Resolution of any coding discrepancies between the 2 researchers involved a consensus process. This allowed the researchers to review the transcript text together, jointly focus on the words, and to best identify concepts raised by the faith leaders.

## 3. Results

The final sample included 26 faith leaders across 23 churches—9 Pastors, 11 Ministry Leaders, and 6 others (i.e., youth advisor, church/health clerk, A.N.G.E.L.). The mean age was 54, and 65% of the sample was female. Christian denominations represented in the sample, included African Methodist Episcopal (3.8%), Baptist (65.3%), Church of God in Christ (23.1%), and Non-denominational (7.7%) faith traditions. Table 1 and Table 2 display the distribution of comfort level discussing sexual behaviors (e.g., anal, oral, and vaginal sex), sexuality, and condom use. Overall, faith leaders felt “comfortable” or “very comfortable” discussing key sexual health behaviors as they relate to HIV risk. Three themes emerged to describe the faith leaders’ perspectives of HIV prevention, sex, and sexuality teachings after a thorough evaluation of focus group discussions. Each theme is described below in detail. Interestingly, participants often self-selected to discuss issues of sexuality akin to homosexuality; hence the terms were sometimes used synonymously.

### 3.1. Restricted by Scripture: “Not a Lot of Room in Our Teachings for Homosexuality”

Scripture is the inspired guidance that orchestrates the worship process and experience and guides the teachings. Faith leaders in our focus groups believed scripture should guide how sex is discussed in church. One participant said:

“We go back in biblical teaching concerning relationships. We feel that you should be married couples before you indulge in sexual activities”.

Across all four focus groups, this proved to be the general consensus, that premarital sex was strongly discouraged. However, almost all acknowledged the reality of unmarried church members still engaging in sex.

Faith leaders described a need to hold the congregation accountable and make them aware of the consequences of sex outside of marriage. Faith leaders discussed nurturing relationships by actively seeking out certain groups, such as the youth, to provide sexual health information and support. Examples of how youth can confidentially call on them for condoms and other advice off church premises were given. Nearly all faith leaders were aware of these premarital sexual relationships. As a result, they believed it was their responsibility to communicate to youth the costs of sex outside of marriage, such as sexually transmitted diseases (STDs).

Participants made a firm distinction about what constitutes a married couple. One participant summarizes their views which were typical of other participants:

“He (God) made a man for a woman and a woman for a man”.

Participants explained that different interpretations of biblical guidance and church doctrines make sexual health teachings difficult, and that church bylaws do not permit conversations about same sex relationships. For context, bylaws (framework) support operations and structure. Some religious denominations have a book of discipline that sets the rules that have been decided at the annual general conference meeting. The churches in this denomination must abide by those rules. These rules have specific instructions on topics such as marriage. One faith leader explained:

“There is not a lot of room in our teaching for homosexuality. Homosexuality in our bylaws is not received”.

Hence, if the topic is discussed, it is limited within the purview of immoral biblical teachings. As illustrated by these quotes, denominational bylaws can perpetuate stigma towards homosexuality, which may limit the ability of a church to address HIV fully.

One participant quoted Mathew 28:20, which reads, “Teaching them to observe (obey) all things whatsoever I have commanded you” as the role of the faith leader in serving congregations. This implies you act like Jesus, especially in how you treat people, and you live your life by the scripture. However, it was universally clear across participants that there were limitations to what is discussed as it relates to same sex relationships and HIV prevention. As one participant noted:

“We have had members who were living with HIV and they have been embraced…but I’m not saying anything about condoms”.

Teaching compassion like Jesus, caring for and providing support to those who are living with HIV, is consistent with biblical scriptures. The faith leader did not hesitate to embrace someone that was ill with compassion but was not willing to say anything about the promotion of condoms because it would exploit perversion, which may inadvertently increase poorer health outcomes. These limitations have a direct impact on church engagement in HIV prevention messaging.

### 3.2. Problematic Silence: “They Don’t Talk”

Participants hinted that the silence and secrecy in congregations around sexual activity in congregations make it difficult to provide HIV prevention and sexual health information. The most notable dangerous depiction of silence was the intersection of poverty and sexual risk behaviors. One pastor explained:

“Many of the people we deal with were brought up in poverty, and it has its own culture. If you’re in a culture where you don’t have a lot of money what do you promote—your, sexuality. I’m a man, you know, I can make babies. That becomes a dominant thing. The more babies you got, the more man you are, you know, in their own thinking”.

Participants were certain that limited employment opportunities and education in this community also affected their ability to openly address HIV and other health problems. Several participants also pointed to young, unwed mothers, as further evidence of the damaging effects of being silent about sexual health teachings. They believed that not discussing sex and sexuality left young people uninformed and less equipped to protect themselves in sexual relationships.

Participants also acknowledged the danger of silence and secrecy among individuals in some of the more rural parts of the Mid-South region (Northern Mississippi, Memphis, TN, North Eastern Arkansas). They believed that adults living with HIV were not always willing to ask for help, as their preference was to keep their HIV status private. As one participant stated:

“But in the rural areas people are very secretive. They don’t talk so that needs to be broken. If you can break the shell of that you can get more education to them. But they’re very secretive and they will not let you into what’s going on with them so that’s one of the things that I picked up on”.

As noted above, enhancing HIV educational efforts is a critical, yet sometimes challenging strategy to encourage more conversations about HIV within closed networks. Another suggested strategy to disrupt the silence surrounding HIV was to personalize HIV when increasing congregational awareness about HIV risk and prevention. The personal relevance of HIV was described as a determinant of support for those infected or affected by HIV. In general, the congregation’s concern about HIV is low if they are not affected personally. One pastor explains:

“Until it hits home you really don’t pay attention until, one of your friends have it, your cousin, somebody. That’s when you want to talk about it”.

As a teaching tool to break the silence, several participants discussed the impact of inviting community members to share their personal testimony and how their message influenced church members to get educated and know their status.

### 3.3. Tackling Multiple Stigmas: “The Church has to Stop Judging and Start Ministering”

There was consensus across focus groups that stigma towards homosexuality was a clear obstacle to fully implement HIV prevention activities. Many endorsed a ‘one man and one woman doctrine’. However, participants recognized the limitations and dangers of this perspective in their efforts to address HIV fully. One participant shared:

“I think that’s part of the reason why the church has been sort of negligent in addressing the issue around HIV because of the church’s stance on homosexuality”.

Homosexuality was considered a sin by all participants. Faith leaders discussed how sins are sometimes ranked. Almost unanimously, participants stated that homosexuality is believed to be the worst sin in the eyes of many churches and many congregations. Faith leaders felt like people needed to resist the temptations of homosexuality, as if sexuality was a personal choice. However, there still remained a level of tolerance and compassion for the individual despite their sexuality or sexual behaviors. One pastor discussed how his congregation had cultivated an atmosphere of tolerance for those members who are different.

Other faith leaders discussed ministerial counseling resources on their church grounds to address some of the hurt, pain, and life issues congregations experience. There was concern that congregants who feel judged for their sexuality may experience threats to their mental wellbeing. One participant explained:

“The highest rate of suicide now is among lesbians and gays and, also veterans. The alienation that one gets, even with the lifestyle, but they’re human”.

Dealing with physical and mental wellbeing facilitates spiritual wellbeing according to faith leaders. Pastors believed it was important as leaders to be cognizant of overall wellbeing and be prepared to minister to the whole person, including the spirit and mind, through worship experiences, small group ministries, and health fairs.

While the issue of stigma toward homosexuality was raised, the impact of stigma on HIV prevention and behavioral risk factors also was discussed in each group. Almost all participants had been involved in or held a church health fair. One woman described common behaviors of congregations as a by-product of the impact of HIV stigma:

“I say the stigma’s there. We will have brochures on sexuality, HIV/AIDS, risk factors, and everybody that will pass by, like, they don’t see it…But if you leave those out and after everything is over you go back they’re all gone because people do want to know, but it’s just the stigma. They don’t want to be seen there”.

It is clear that congregants are eager to receive HIV related information but are reluctant to openly express their desires for more information because of the stigma associated with HIV. Seeking information about HIV may raise unwarranted judgments about congregants’ sexual behaviors. Another participant perpetuated a common belief held by faith leaders and one that is often disseminated to congregations and communities. She explained to the group:

“The guys are not going to come out and talk openly about it because of the stigma that’s attached to the homosexual behavior that’s going on in the jails and it is a lot of it, believe me. I mean our young men—African American males are participating in this behavior big time in the jails. Then they come out of the jails, get back with their girlfriends and that’s why you see the numbers”.

These salient examples shared by faith leaders illustrate the influence of stigma associated with HIV in faith contexts. Although faith leaders are acknowledging the impact of stigma on health-seeking behaviors, they were unaware of interventions or messages that would decrease stigma. Thus, faith leaders are in agreement that leaders in the church would benefit greatly from education to combat stigma.

## 4. Discussion

There is a growing body of literature in faith-based HIV research that suggests the field has moved far beyond simplistic conversations of abstinence only, no sex before marriage, and homosexuality as a sin. However, the vast majority of this work has been conducted in more progressive cities outside of the Deep South, such as Baltimore, Los Angeles, Kansas City, New York City, and Philadelphia [6,9,33,34,35].

Church leadership faces its own unique concerns in the South, which is traditionally thought to be more conservative than other regions of the country [36]. The reality remains that there are major challenges with addressing the topics of sex, sexuality, and HIV in some Southern churches that might not be as prevalent outside of the South. Taking a more culturally congruent approach to prevention is certainly suggested to engage churches; however, conversations about sex and sexuality cannot be ignored, especially in the South, where such topics can be a stigmatizing force. While promoting abstinence as the ideal, faith leaders recognized the reality of premarital sexual activity and same sex relationships occurring among congregational members. Likewise, church leaders in our study had compassion for and embraced those living with HIV, despite their sexuality and previous known sexual risk behaviors. Acknowledgment of the importance of educating congregations about HIV and no longer remaining silent has implications for how the conversation about HIV prevention moves forward.

Homosexuality was used synonymously with sexuality among participants, which implies little distinction between sexual orientation and the ability to simply experience sexual feelings. Faith leaders self-selected the term homosexuality over sexuality. This is congruent with other studies with faith leaders framing sexuality to a very limited definition [20]. Nonetheless, faith leaders were in conflict with reconciling biblical teachings of homosexuality. Faith leaders practice compassion and tolerance but adhere to and abide by denominational bylaws condemning homosexuality. This finding reflects the existence and persistence of opposing responses that some faith leaders have. This persistent struggle of faith leaders is not uncommon to the feelings that some gay, lesbian, and bisexual individuals feel. Particularly among black men who have sex with men, negative religious experiences create internalized distress [14,37,38].

There are competing concerns of how best to integrate sensitive topics. The nature and direction of what is said to church members suggest that faith leaders are comfortable saying homosexuality is a sin, yet there is still a place for lesbian, gay, bisexual, transgender and queer (LGBTQ+) persons in the church. Understanding how this dual reality is perceived by and impacts Black gay men needs to be explored. Ward (2005) uncovers dimensions of Black gay men’s connectivity to spirituality and departure from religion as one possible explanation [39]. He describes how hyper-masculinity is constructed and may be a by-product of homophobia in the church, placing men at risk for HIV. On the other hand, researchers have examined the range of health activities in churches and have found HIV-related stigma reduction is couched or incorporated as part of awareness and advocacy efforts [33]. The utility of using similar approaches with sexuality also warrants further investigation.

Our findings demonstrate that interpretations of biblical scripture and selective use of scripture affects how faith leaders engage in specific conversations about sexuality and sex altogether. Faith leaders use scripture to guide their messaging, which may unintentionally lead to discussions about sexuality that have severe implications for perpetuating stigma. Prior research has shown that when stigma around their sexuality is present in churches, Black gay and bisexual men might suffer from social isolation and an unwillingness to seek guidance from their church leadership [14]. Stigma around sexuality has also been known to ignite fears of losing congregants among some pastors [12,34]. A church case study in the Midwest, assessing barriers that clergy experienced in starting and sustaining an HIV ministry, found that the primary barrier was views regarding sexuality [40]. Likewise, discussions around the challenges of addressing sexuality in its entirety did occur in our focus groups. Moving forward, how do we help faith leaders effectively address sexuality and simultaneously respect their doctrine?

One strategy to increase readiness to address same sex relationships is for clergy to initiate discussions about how people who do not feel accepted and judged has implications on their mental wellbeing. This was a salient finding in one of our discussion groups that should not be ignored. The minority stress model [41] explains how the intersection of discrimination, prejudice, and stigma among lesbian, gay, and bisexual individuals, may create a hostile and stressful environment that leads to worse mental health. Given their salient role in counseling mind, body, and spirit, clergy can gradually incorporate HIV-related prevention messages along this vein. Moreover, research suggests Black men who have sex with men use church as a means to cope and reconcile sexuality through worship and prayer for the forgiveness of sins [42,43]. Tailoring interventions, with the help of church leadership and members of the congregations, could be one way to help alleviate stigma and help to decrease social isolation among Black gay and bisexual men [44]. Facilitating readiness to acknowledge the benefits of coping strategies in spiritual counseling and ministerial work also should be considered in clergy development and theological training.

Another strategy is to take a risk reduction approach to sexual health within churches. Some faith leaders acknowledged premarital sex among their congregants and discussed efforts to reduce their risk of contracting HIV or having an unplanned pregnancy. The effectiveness of risk reduction approaches has been well documented among several at-risk sub-populations [45,46]. Faith leaders were not as rigid of how their interpretations of the bible were communicated, as many assisted with STD prevention and supplied sexual health resources for subgroups of the church. They discussed the importance of holding the congregation accountable.

Considering the entire continuum of HIV prevention and care, the roles of faith leaders are not only critical among HIV prevention messaging, but also for people living with HIV (PLWH). Given the high participation of church attendance in the southern region, it is exceedingly probable that PLWH attend worship services regularly without disclosing their HIV status to fellow church members [47]. Clergy could reinforce the importance of medication adherence and retention in medical care during worship services. Health ministries could facilitate linkage to medical care or HIV testing events [48,49]. Therefore, resources to increase compassion and church support for those infected and affected could benefit many congregations and reduce HIV-related stigma in churches [19,50].

### Study Limitations

We chose to use focus groups as opposed to individual interviews because of the inter-faith connections this community exudes and to examine group norms. However, the topic of sexuality and sexual health may have been too sensitive to discuss in focus groups. We diversified the groups to minimize any discomfort and gave participants the opportunity to refrain from answering certain questions. This allowed for a thought-provoking group discussion across denominations and leadership levels. Faith leaders self-selected to participate, thus may view issues of sexuality differently than non-participants given their familiarity with HIV risk behaviors. The possible impact of HIV framing should be considered within the interpretation of the qualitative findings. Declines in physical attendance and church participation over the years may have limited the findings as faith leaders discussed variations in church-based activities but not those online. Sampling is another limitation as faith leaders were mostly recruited from a convenience professional and personal network of Christian churches from one city in a very conservative U.S. Southern state. Finally, as with all qualitative studies, the small sample limits generalizability. Yet Memphis has been compared to other urban southern cities like Atlanta and New Orleans with similar new HIV infection rates and religious influence.

## 5. Conclusions

This study described how HIV prevention, sexuality, and sexual health teachings are addressed from the perspective of Black faith leaders ministering in the South. Given the differing perceptions of clergy and faith leaders in the conservative South, it is often assumed that inclusiveness and compassion for people living with HIV and BMSM are absent in churches. Our findings demonstrate that this is not the case, but rather there are nuanced approaches in the delivery of teachings across faith leaders. Future studies might consider the ways in which biblical scripture is interpreted and selected to promote messaging and implications of denominational bylaws in both virtual and traditional worship spaces. Working in tandem with all LGBTQ+ communities and faith leaders to dissect and craft messages will reach those most at risk for acquiring HIV and those living with HIV to provide a truly inclusive and supportive environment. This becomes particularly important as nationwide jurisdictions develop local Ending the HIV Epidemic Plans integrating strategies to address stigma and homophobia within the four pillars.

To situate examples of our findings in the context of relevant EtHE Initiative pillars, we propose partnerships engaging faith communities to consider the following recommendations:

1. Diagnose Pillar—acknowledge premarital sex among congregational members and offer regular HIV testing on church premises to normalize testing and educate members on knowing their HIV status.
2. Prevent Pillar—recognize the possibility of congregational members engaging in condomless sex and substance use and offer HIV prevention information on pre-exposure prophylaxis and syringe services programs.
3. Treat Pillar—deal with the whole person and promote physical, mental, and spiritual health by encouraging congregational members to access wellness services to improve health outcomes effectively.
4. Respond Pillar—maintain transparency with congregations and the surrounding church community of potential outbreaks and become familiar with resources to get needed treatment and prevention services to those affected.

Disentangling our primary findings regarding the stringency of bylaws, suffering in silence, and stigmatization as potential threats to HIV prevention and applying them directly to the EtHE pillars, we may inform the development of effective interventions in churches and local Ending the HIV Epidemic implementation plans.

## Figures and Tables

**Table 1:** Distribution of faith leaders’ comfort level discussing sexual health topics with youth (*n* = 26).

Topic	% (*n*)			
	Not Comfortable	Somewhat Comfortable	Comfortable	Very Comfortable
Homosexuality	7.7 (2)	7.7 (2)	26.9 (7)	57.7 (15)
Anal sex	15.4 (4)	15.4 (4)	30.8 (8)	34.6 (9)
Oral sex	15.4 (4)	15.4 (4)	26.9 (7)	38.5 (10)
Vaginal sex	3.8 (1)	11.5 (3)	34.6 (9)	50.0 (13)
Condoms	7.7 (2)	11.5 (3)	23.1 (15)	57.7 (15)

**Table 2:** Distribution of faith leaders’ comfort level discussing sexual health topics with adults (*n* = 26).

Topic	% (*n*)			
	Not Comfortable	Somewhat Comfortable	Comfortable	Very Comfortable
Homosexuality	-	7.7 (2)	30.8 (8)	61.5 (16)
Anal sex	7.7 (2)	23.1 (6)	26.9 (7)	42.3 (11)
Oral sex	7.7 (2)	23.1 (6)	26.9 (7)	42.3 (11)
Vaginal sex	-	7.7 (2)	42.3 (11)	50.0 (13)
Condoms	-	11.5 (3)	26.9 (7)	61.5 (16)
